# Telerehabilitation Using Fitness Application in Patients with Severe Cystic Fibrosis Awaiting Lung Transplant: A Pilot Study

**DOI:** 10.1155/2021/6641853

**Published:** 2021-02-26

**Authors:** Aimee M. Layton, Andrew M. Irwin, Erin C. Mihalik, Emily Fleisch, Claire L. Keating, Emily A. DiMango, Lori Shah, Selim M. Arcasoy

**Affiliations:** ^1^Department of Pediatrics, Division of Pediatric Cardiology, Columbia University, New York, NY, USA; ^2^Vagelos College of Physicians and Surgeons, Columbia University, New York, NY, USA; ^3^Department of Applied Physiology, Teachers College, Columbia University, New York, NY, USA; ^4^Department of Rehabilitation and Regenerative Medicine, Columbia University, New York, NY, USA; ^5^Division of Pulmonary, Allergy and Critical Care Medicine, Department of Medicine, Columbia University Medical Center, New York, NY, USA; ^6^Lung Transplant Program New York Presbyterian Hospital, Columbia University Medical Center, New York, NY, USA

## Abstract

**Purpose:**

The purpose of this study was to pilot a home-based pulmonary rehabilitation (PR) program administered via a telemedicine approach using a combination of fitness application and self-selected activity in lung transplant candidates with cystic fibrosis (CF).

**Methods:**

We recruited adult patients with CF. The main outcome was adherence, measured by number of sessions completed in 12 weeks. Secondary outcomes were adverse events, six-minute walk distance (6MWD), and dyspnea. Participants were provided a personalized exercise program and equipment including a fitness application that provided exercise videos, recorded exercise time, and corresponding heart rate. We reviewed data daily and provided text messages with feedback. We compared our study outcomes to a retrospective data set of CF patients who participated in a 24-session outpatient hospital-based PR program. Data presented as mean ± standard deviation.

**Results:**

Eleven patients participated in the home PR program, 45% female, age 33 ± 7 years, FEV1 27 ± 5% predicted. Sessions completed were 19 ± 12 home-based PR vs. 9 ± 4 hospital-based PR, *p* = .03. Fifty percent of the home-based group completed ≥24 sessions in 12 weeks versus 0% of the hospital-based patients (*p* = .03). There were no adverse events during exercise. Completers of the home-based program demonstrated a clinically meaningful lower decline in 6 MWD than noncompleters (6MWD −7 ± 15 vs. −86 ± 108 meters). Only one participant performed a post 6 MWD in the hospital-based PR.

**Conclusion:**

Patients with severe CF demonstrated adherence to home PR delivered using fitness application and self-selected activity with no adverse events. This program style may be a viable solution for telerehabilitation in severe CF and is particularly relevant in the COVID era.

## 1. Introduction

Maintaining a level of physical vigor prior to lung transplantation is important in improving postoperative outcomes [1]. Patients with severe cystic fibrosis (CF) suffer from poor exercise tolerance and physical frailty that correlates with disease severity [2–5]. Traditional pulmonary rehabilitation (PR) improves exercise tolerance and physical frailty in severely ill CF patients, including those who may be eligible for lung transplant [6]. However, there are limitations to providing in-person center or hospital-based PR in the CF population [7].

Participation in a hospital-based pulmonary rehabilitation (PR) program is problematic for patients with CF due to the group setting leading to increased risk of infection, commute to the center, work/family time conflicts, and its traditional design for a largely elderly population [7]. The recent pandemic has accelerated the need for a hybrid or entirely home-based PR program option for many individuals [8]. Home-based exercise programs have been found to be safe and effective in pediatric and more mild to moderately ill adult CF populations [9, 10] but have not been evaluated for the severely ill adult CF population. Therefore, a home-based PR program delivered via telemedicine could provide adults with severe CF awaiting lung transplantation a more feasible opportunity to enroll in PR and ultimately maintain a level of physical vigor needed for optimal postsurgical outcomes.

Recent developments in technology have led to the ability to stream exercise classes both live and prerecorded to one's television or smart phone. Given the relatively young age of the adult CF population, we felt a commercial fitness application designed for such demographic would have better adoption than applications designed for the traditional PR population. Traditional applications designed for PR have focused on the chronic obstructive pulmonary disease (COPD) population and adults ≥65 yrs [11]. We selected the “Peloton” application or “app” for this study. The Peloton app provided a dashboard for the study team to monitor heart rate (HR) throughout exercise and track classes taken and exercise time. As Peloton app usages and our monitoring were asynchronous, we allowed subjects flexibility of both timing and indoor/outdoor locations for their home-based exercise. We asked they wear their HR monitors and record HR and exercise time via either the Peloton app or the health app on their phone.

The purpose of this study was to pilot a home-based PR program administered via telemedicine in lung transplant candidates by using a combination of fitness application and self-selected activity. Our main outcome measure was adherence to the program quantified by the number of exercise sessions completed within 12 weeks. We hypothesized that patients would demonstrate greater adherence to the home-based PR than that shown in historic data from the hospital-based outpatient PR program. Secondarily, we sought to determine if the program design was safe and effective in improving shortness of breath and exercise capacity.

## 2. Materials and Methods

We recruited adult patients with CF from the Lung Transplant Program and Cystic Fibrosis Center to participate in the telerehabilitation program. We also performed a retrospective chart review of adult patients with CF who had enrolled in an outpatient hospital-based PR program at New York-Presbyterian Hospital from January 1, 2015, to September 30, 2019. We compared the number of sessions in 12 weeks and number of patients who completed a full program, defined as at least 24 sessions of this population to our study population. The Columbia University Medical Center human protection committee (IRB) approved the study.

Prior to starting the program, all patients performed basic spirometry [12], a general self-efficacy scale [13], international physical activity questionnaire [14], a six-minute walk test (6 MW) [15], and a Cardiopulmonary Exercise Test (CPET) [16]. The CPET was performed on a cycle ergometer using a 10-watt ramping protocol with breath-by-breath gas exchange measurements, full 12-lead electrocardiogram, and pulse oximetry (VMAX ENCORE, Vyaire, Yorba Linda, CA). The complete protocol is found in a prior publication [17]. Patients were excluded from the study if they demonstrated any of the following with exercise: severe oxyhemoglobin desaturation (SpO_2_) at rest or during exercise (SpO_2_ ≤ 85%), hypercapnia (ETCO_2_ ≥ 60 mmHg), symptoms of dizziness, headache, nausea, or chest pain during the exercise, required > 6 L/min of supplemental oxygen during the 6 MW test, and significant ST depression or elevation with exercise or significant ectopy [16].

### 2.1. Measurements

Our main outcome was adherence to exercise measured by number of sessions completed in 12 weeks. Sessions mimicked traditional PR and consisted of a prescription of aerobic exercise, strength training, and stretching. A full program of PR was at least 24 sessions in ≤12 weeks. Secondary outcomes were number of individual workouts (i.e., cycling for 20 mins was one workout, strength training for 10 mins was another workout), adverse events, increase in six-minute walk distance (6MWD), and decrease in dyspnea with activity measured by the UCSD shortness of breath questionnaire [18]. Patients recorded, on an exercise log provided, their nadir pulse oximetry reading during exercise, shortness of breath on a 0-10 modified Borg scale, and any symptoms they experienced during exercise. The Peloton app recorded exercise time and HR throughout exercise to a dashboard that the study team could access.

### 2.2. The Program

The program was considered semisupervised as patients did not have someone from the study team watching them exercise, but rather the study team asynchronously reviewed the data daily (HR and exercise time via Peloton app dashboard) and texted feedback to the patient. Patients were instructed to text back any feedback they wanted to provide regarding the workout, such as symptoms, questions, or comments. We sent educational material and a personalized exercise plan to the patient weekly via email and reviewed data from the exercise logs weekly, which included self-reported nadir SpO_2_ with exercise, highest Borg score, and any symptoms expressed. The personalized exercise plan included HR range targets and Borg scale number for a given exercise intensity based on their CPET results and a minimum amount of time for each exercise modality. The study team modified the exercise prescriptions weekly to meet the patient's needs. We instructed patients to record the exercise time and HR by having the Peloton app on while they either followed an exercise video via the app or performed a self-selected activity such as walking or hiking.

**Figure 1:**
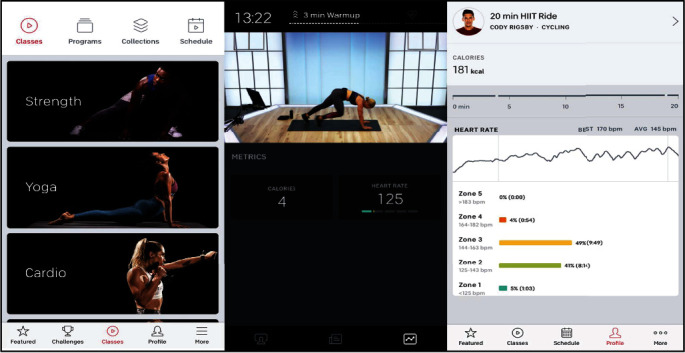
Example of Peloton application user interface.

Patients took live classes with the Peloton app or could access a library of workout videos to follow along with varying length (5 mins to 60 mins) and intensity (easy to very difficult). The videos consisted of cycling, treadmill walking, yoga, strength training, stretching, outdoor walking or running, dance videos, body weight exercises, and plyometric exercises. The study team instructed patients on how to modify the activities if the intensity felt too strenuous.  Figure 1 demonstrates how a Peloton class appeared to the patient with the coinciding data for the study team.

**Figure 2:**
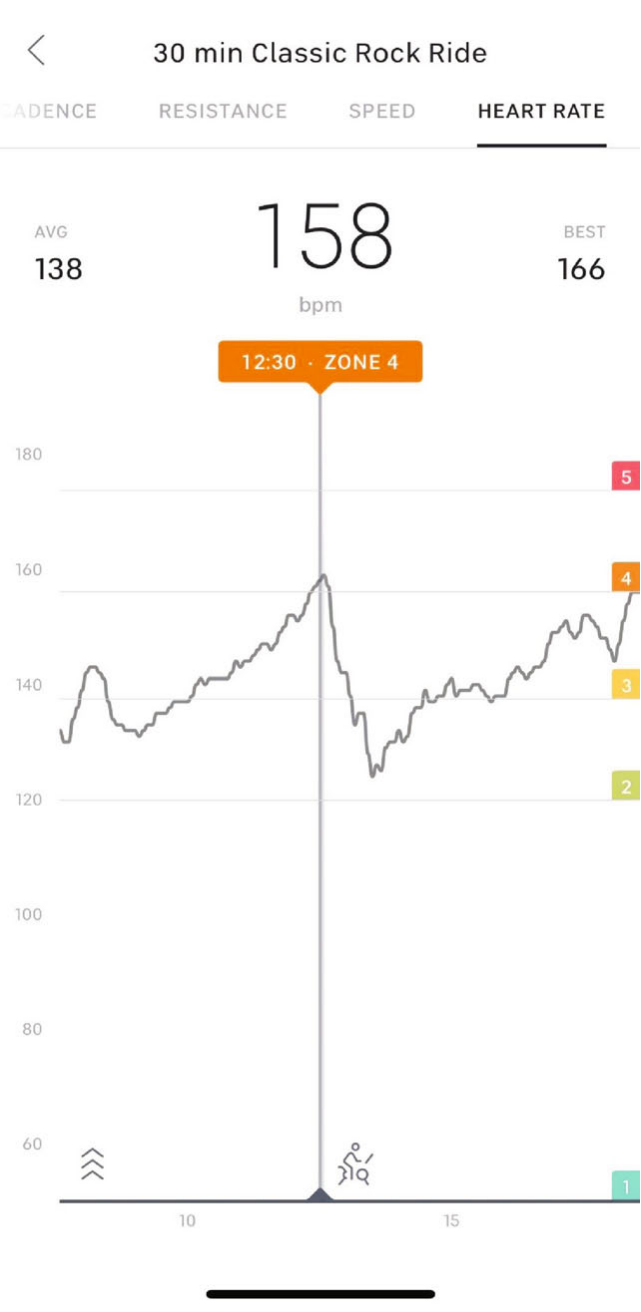
Example of heart rate data input from Bluetooth strap.

A Bluetooth-enabled heart rate monitor was used to track HR (Peloton, New York, NY). The software graphed the HR beats per minute provided (simple log not electrocardiogram signal) throughout the entirety of the exercise (Figure 2).

The study member could scroll along the activity timeline and see at any given moment the heart rate collected. The application also provided an average of the heart rate for the entirety of the activity and the maximum heart rate achieved.

### 2.3. Analysis

We used SPSS v24.0 (IBM, Armonk, NY) for data analysis. Significance was set a priori at *p* ≤ .05. We expressed continuous variables as medians and interquartile range (IQR) and categorical variables as frequency and percentiles. Differences in baseline characteristics and demographics between the home PR group and hospital based seen in Table 1 were tested using a nonparametric testing which was a Chi square analysis for categorical variables and Mann-Whitney *U* analysis for continuous variables. We tested difference in our main outcome variable, the number of sessions completed, and our secondary outcome variable, total number of workouts performed, between the home-based PR and hospital-based PR using an independent *t*-test analysis. Frequency of program completion between groups was analyzed using the nonparametric test Chi-square analysis. We tested correlations between baseline characteristics and number of sessions completed in the home-based exercise group using a Spearman correlation. Changes in six-minute walk test and dyspnea scores between groups could not be tested due to large number of hospital-based PR patients dropping out of PR prior to completing a post 6 MW test or UCSD shortness of breath questionnaire.

## 3. Results

Twenty pre-lung transplant candidates with CF visited the center during our recruitment period. The study team approached 18 of those patients. Two patients were not approached due to scheduling conflicts. Of the 18 patients approached, five were not interested in participating. One patient was screened via CPET and met exclusion criteria based on hypercapnia with exercise (ETCO2 ≥ 60 mmHg). One of the 18 patients received a lung transplant after consenting but prior to starting the program. Table 1 describes the baseline demographics and clinical characteristics for the final study population. Eleven patients participated in home PR. Hospital-based outpatient PR group included 8 CF patients. There were significantly more women in the outpatient hospital PR group than in the home exercise group (*p* = .01).

**Figure 3:**
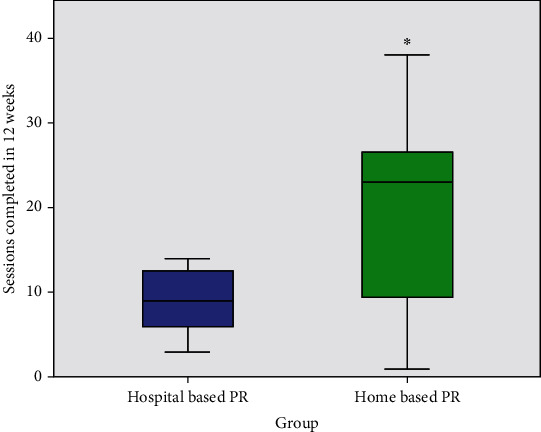
Sessions completed. PR: pulmonary rehabilitation. ^∗^Significant differences in sessions completed between groups, *p* = .04.

Five of the eleven participants completed at least 24 sessions of home exercise in 12 weeks; this was significantly more than the outpatient hospita-based program (*p* = .03) (Table 2). Patients in the home exercise group completed significantly more sessions in 12 weeks than the outpatient hospital-based PR group (*p* = .03) (Table 2, Figure 3).

There was high variability in the number of sessions completed in the home-based PR group when compared to the hospital-based PR group (Figure 3). The patients' history of physical activity before starting the program correlated with a high number of sessions performed during the program (*r* = .66, *p* = .03) and workouts completed (*r* = .65, *p* = .03). The total number of different workouts was not significant between groups (Table 2) (*p* = .53). Three participants (27% of the study population) chose to purchase the application upon study completion and continued using it on their own. Self-selected non-app activities consisted of hiking and outdoor walking.

There were no serious adverse events during exercise. The most frequently reported symptom with exercise was muscle fatigue (3 out of 11 patients). Other common symptoms were cough or wheezing (3 out of 11 patients), shortness of breath (1 out of 11 patients), and headache after exercise, which quickly subsided (2 out of 11 patients). One patient notified the study team of chest discomfort during exercise after a session, which was ultimately determined to be from an acute infection. Two patients expressed lightheadedness with exercise that was associated with low blood sugar. The study team instructed the patient make sure their finger stick glucose was between >100 mg/dL and < 250 mg/dL prior to exercise. One patient was experiencing frequent hemoptysis. His physician cleared him for exercise, but during one session, he experienced minor hemoptysis while performing push-ups and informed his doctor and the study team immediately. His doctor told him to take 2 weeks off from exercise, and he received physician clearance prior to resuming the exercise program. The study team instructed the patient not to perform prone exercises.

Patients who did not complete at least 24 sessions within 12 weeks of the home-based PR program (*n* = 6) demonstrated a clinically meaningful decline in 6 MWD [19] of mean −86 ± 108 meters (standard error). Whereas those who completed the program remained stable, mean −7 ± 33 meters (standard error). Few patients in the outpatient hospital-based PR program performed post 6 MW tests (*n* = 2); therefore, an analysis could not be performed between groups. Not all patients sent back the UCSD shortness of breath questionnaires but of those that did (*n* = 7), scores decreased with the home-based PR program from 49 ± 26 to 43 ± 14, *p* = .51. This decrease met the threshold for a clinically meaningful difference [20]. Only one patient from the hospital-based program completed the UCSD post-PR questionnaire.

## 4. Discussion

A semisupervised home pulmonary rehabilitation program in pre-lung transplant adult patients with CF resulted in significantly better adherence than traditional hospital-based pulmonary rehabilitation. Similar to other telerehabilitation research in lung transplant candidates [21], no serious adverse events resulted from the exercise performed. Patients communicated symptoms that needed further evaluation effectively with the study team and their referring physician. Patients who completed the home-based PR program demonstrated clinically meaningful differences in 6 MWD and shortness of breath.

There was high variability in the number sessions completed in the home-based PR group. Such variability reflected a cohort who exceled in a more flexible environment and a cohort who may thrive in a center-based program. Similar to work in other patient populations [22], our results demonstrated that a history of being physically active corresponded with better adherence. Therefore, patients with no history of being physically active may have better adherence to a hybrid-structured program or traditional in person PR program, where they can begin by exercising at the center, then transition to home exercise or exercise entirely at a center.

The program we created was unique because of the combination of self-selected activity and use of a fitness application designed for the general population. The study team monitored compliance and exercise intensity using a Bluetooth-enabled heart rate monitor and the patient's smart phone. By using such technology, the study was able to maximize the flexibility of home-based exercise at a low cost (HR monitor was $50, application was $12/month). Thus, patients could exercise outdoors and at varying times. Prior research in home-based exercise in the CF population found compliance to be a major barrier in effectiveness [23]. In the pediatric CF population, when activity included recreation elements and the ability to choose between different activities, compliance and adherence were improved [23, 24]. The combination of self-selected recreational activity and a large selection of classes to select (>1,000 classes to select from) likely contributed to increased compliance based on this prior work.

Home-based pulmonary rehabilitation produced similar results as the hospital-based pulmonary rehabilitation program. These results are consistent with research in other populations such as COPD [25–27]. A large randomized controlled trial is under way to investigate differences in clinical outcomes and the cost of a home-based telerehabilitation [28]. Unfortunately, this study does not include patients under 40 years of age. Thus, few patients with CF will be represented in the study population. Findings from our study indicate that the 18-40 year-old demographic may also demonstrate promising results and potentially greater benefit from a telerehabilitation approach. We hope future research will consider incorporating this patient population into their cohort.

This work piloted the use of a fitness application designed and commercially available for the general population in a severely ill patient population. When researching available applications for home-based PR, we found all pulmonary rehabilitation applications were designed for the COPD and/or older lung disease population [11, 21]. The demographic of CF is a younger population [29]. We were concerned the CF population would not relate to the videos and style of exercise in the available PR apps. Therefore, we decided to trial a fitness application designed for their age group and designed to be entertaining. We found the Peloton application provided enough low intensity exercise that patients were able to perform the classes or modify to their fitness level. The study team did instruct patients on how to modify exercise such as taking longer recovery or a lower resistance than intensity suggested by the instructor. There was also a large library of classes to choose from with varying types of exercise from resistance training to outdoor walking, which lends to greater engagement by the user. There is a large array of music genres that coincide with the classes, which many other pulmonary rehabilitation-based applications did not include. Additionally, the patients were able to work out at the same time as other individuals on the app if they chose, which provided the community setting that is lacking in many telerehabilitation programs. The negative of using this fitness application was the lack of education and inability to integrate pulse oximetry into the platform. We worked around this limitation by emailing the educational component to the participants weekly and having them provide us their pulse oximetry readings in their journal. However, participants did not always comply with answering the education questions and not all returned their journals every week.

### 4.1. Limitations of the Study

Limitations of this work include the lack of randomization between the home-based group and hospital-based group. This can lead to selection bias in these data, as the hospital-based group were all women. Despite the lung function of the groups being similar, not all of the hospital-based patients were being evaluated for a lung transplant at the time of their pulmonary rehabilitation program. Future directions of this work will be to trial this study design in a larger and randomized population. The inability to acquire post 6 MW data in people who dropped out or quarantined from COVID-19 was also a limitation. Future work will consider the use of tests performed remotely such as the 30-second sit-to-stand as a functional assessment outcome rather than 6 MW.

### 4.2. Future Directions

The lack of real-time supervision may be considered a limitation of the program [30]; however, we consider this a strength. The low reimbursement rate of pulmonary rehabilitation has caused many programs to close, long wait lists, and fewer programs available for patients within a reasonable distance to their house [31]. There is a need to provide pulmonary rehabilitation to more people with fewer resources. Knowledge that a semisupervised model is safe and effective would allow some programs to use this design in a hybrid approach to pulmonary rehabilitation. Future directions of this work will be to use this program design in conjunction with an in-person program to allow more patients to be seen in a finite amount of time.

## 5. Conclusion

Patients with severe CF demonstrated adherence to semisupervised home PR delivered using a fitness app and self-selected activity with no adverse events. This program style may be a viable solution for telerehabilitation in severe CF and may be particularly relevant in the era of the COVID-19 pandemic. Further research can elucidate possible benefits of home PR in lung transplant outcomes.

## Figures and Tables

**Table 1:** Patient demographics and baseline clinical characteristics.

Characteristic	Home PR (*n* = 11)	Outpatient hospital PR (*n* = 8)	*p* value
Age, years	30 (10)	29 (7)	0.12
Female	5 (46%)	8 (100%)	*0.01*
BMI, kg/m^2^	19.7 (5.4)	20.0 (5.8)	0.71
FeV1 (L)	.88 (.49)	.95 (.51)	0.84
FeV1 (% predicted)	26 (6)	29 (14)	0.28
O_2_ requirements during exercise, L/min	2 (4)	1 (4)	0.78
Pre 6-minute walk test, meters	412 (106)	394 (130)	0.81
Pre UCSD dyspnea	43 (34)	58 (45), *n* = 7	0.77
GSE	27 (10), *n* = 8	NA	
IPAQ	165 (784)	NA	

Medians and (interquartile range). BMI: body mass index; FeV1: forced expiratory volume during 1 second; UCSD: University of California San Diego (Shortness of Breath Questionnaire); PR: pulmonary rehabilitation, GSE: General Self-Efficacy Questionnaire; IPAQ: International Physical Activity Questionnaire.

**Table 2:** Adherence to the exercise program.

Characteristic	Home PR (*n* = 11)	Hospital PR (*n* = 8)	*p* value
Subjects to complete program^∗^	5 (46%)	0 (0%)	*0.03*
Sessions in 12 weeks, days	19 ± 12	9 ± 4	*0.03*
Total # of workouts^∗∗^	31 ± 28	27 ± 11	0.53

^∗^Completion of program defined as completing 24 exercise sessions in 12 weeks. ^∗∗^Workout is defined as a single component of total exercise program (i.e., 3 workouts = 1 exercise session). A workout may include resistance training, aerobic training, stretching, and yoga.

## Data Availability

Due to patient privacy, the data is restricted.

## References

[1] Hayes K., Hodgson C. L., Pellegrino V. A. (2018). Physical function in subjects requiring extracorporeal membrane oxygenation before or after lung transplantation. *Respiratory Care*.

[2] Layton A. M., Armstrong H. F., Baldwin M. R. (2017). Frailty and maximal exercise capacity in adult lung transplant candidates. *Respiratory Medicine*.

[3] Ferguson N., Proud D., Bridges C., Duckers J. (2016). Cystic fibrosis: detecting frailty in an outpatient clinic. *Healthy Aging Research*.

[4] Singer J. P., Diamond J. M., Gries C. J. (2015). Frailty phenotypes, disability, and outcomes in adult candidates for lung transplantation. *American Journal of Respiratory and Critical Care Medicine*.

[5] Wilson M. E., Vakil A. P., Kandel P., Undavalli C., Dunlay S. M., Kennedy C. C. (2016). Pretransplant frailty is associated with decreased survival after lung transplantation. *The Journal of Heart and Lung Transplantation*.

[6] Li M., Mathur S., Chowdhury N. A., Helm D., Singer L. G. (2013). Pulmonary rehabilitation in lung transplant candidates. *The Journal of Heart and Lung Transplantation*.

[7] Hayton C., Clark A., Olive S. (2013). Barriers to pulmonary rehabilitation: characteristics that predict patient attendance and adherence. *Respiratory Medicine*.

[8] Elbeddini A., Tayefehchamani Y. (2021). Amid COVID-19 pandemic: challenges with access to care for COPD patients. *Research in Social & Administrative Pharmacy*.

[9] del Corral T., Cebrià i Iranzo M. À., López-de-Uralde-Villanueva I., Martínez-Alejos R., Blanco I., Vilaró J. (2018). Effectiveness of a home-based active video game programme in young cystic fibrosis patients. *Respiration*.

[10] de Jong W., Grevink R. G., Roorda R. J., Kaptein A. A., van der Schans C. P. (1994). Effect of a home exercise training program in patients with cystic fibrosis. *Chest*.

[11] Rassouli F., Boutellier D., Duss J., Huber S., Brutsche M. H. (2018). Digitalizing multidisciplinary pulmonary rehabilitation in COPD with a smartphone application: an international observational pilot study. *International Journal of Chronic Obstructive Pulmonary Disease*.

[12] Graham B. L., Miller M. R., Barjaktarevic I. Z. (2019). Standardization of spirometry 2019 update. An official American Thoracic Society and European Respiratory Society technical statement. *American Journal of Respiratory and Critical Care Medicine*.

[13] Luszczynska A., Scholz U., Schwarzer R. (2005). The general self-efficacy scale: multicultural validation studies. *The Journal of Psychology*.

[14] Hagstromer M., Oja P., Sjostrom M. (2006). The International Physical Activity Questionnaire (IPAQ): a study of concurrent and construct validity. *Public Health Nutrition*.

[15] Salzman S. H. (2009). The 6-min walk test: clinical and research role, technique, coding, and reimbursement. *Chest*.

[16] Balady G. J., Arena R., Sietsema K. (2010). Clinician’s guide to cardiopulmonary exercise testing in adults: a scientific statement from the American Heart Association. *Circulation*.

[17] Layton A. M., Armstrong H. F., Kim H. P., Meza K. S., D'Ovidio F., Arcasoy S. M. (2017). Cardiopulmonary exercise factors predict survival in patients with advanced interstitial lung disease referred for lung transplantation. *Respiratory Medicine*.

[18] Eakin E. G., Resnikoff P. M., Prewitt L. M., Ries A. L., Kaplan R. M. (1998). Validation of a new dyspnea measure: the UCSD Shortness of Breath Questionnaire. *Chest*.

[19] Shoemaker M. J., Curtis A. B., Vangsnes E., Dickinson M. G. (2013). Clinically meaningful change estimates for the six-minute walk test and daily activity in individuals with chronic heart failure. *Cardiopulmonary physical therapy journal*.

[20] Kupferberg D. H., Kaplan R. M., Slymen D. J., Ries A. L. (2005). Minimal clinically important difference for the UCSD Shortness of Breath Questionnaire. *Journal of Cardiopulmonary Rehabilitation*.

[21] Singer J. P., Soong A., Bruun A. (2018). A mobile health technology enabled home-based intervention to treat frailty in adult lung transplant candidates: a pilot study. *Clinical Transplantation*.

[22] Ormel H. L., van der Schoot G. G. F., Sluiter W. J., Jalving M., Gietema J. A., Walenkamp A. M. E. (2018). Predictors of adherence to exercise interventions during and after cancer treatment: a systematic review. *Psychooncology*.

[23] Bar-Or O. (2000). Home-based exercise programs in cystic fibrosis: are they worth it?. *The Journal of Pediatrics*.

[24] Schneiderman-Walker J., Pollock S. L., Corey M. (2000). A randomized controlled trial of a 3-year home exercise program in cystic fibrosis. *The Journal of Pediatrics*.

[25] Holland A. E., Mahal A., Hill C. J. (2016). Home-based rehabilitation for COPD using minimal resources: a randomised, controlled equivalence trial. *Thorax*.

[26] Güell M. R., de Lucas P., Gáldiz J. B. (2008). Home vs hospital-based pulmonary rehabilitation for patients with chronic obstructive pulmonary disease: a Spanish multicenter trial. *Archivos de Bronconeumología*.

[27] Strijbos J. H., Postma D. S., van Altena R., Gimeno F., Koëter G. H. (1996). A comparison between an outpatient hospital-based pulmonary rehabilitation program and a home-care pulmonary rehabilitation program in patients with COPD: a follow-up of 18 months. *Chest*.

[28] Cox N. S., McDonald C. F., Alison J. A. (2018). Telerehabilitation versus traditional centre-based pulmonary rehabilitation for people with chronic respiratory disease: protocol for a randomised controlled trial. *BMC Pulmonary Medicine*.

[29] Sanders D. B., Fink A. K. (2016). Background and epidemiology. *Pediatric Clinics of North America*.

[30] Moy M. L. (2019). Not all home-based exercise programs are home-based pulmonary rehabilitation programs. *American Journal of Respiratory and Critical Care Medicine*.

[31] Garvey C., Novitch R. S., Porte P., Casaburi R. (2019). Healing pulmonary rehabilitation in the United States. A call to action for ATS members. *American Journal of Respiratory and Critical Care Medicine*.

